# Free Medical Schools

**Published:** 1849-11

**Authors:** 


ARTICLE IV.

*Free Medical Schools.*—[From the Boston Med. & Surg. Jour.]—The gentleman who has the credit of being the originator of the American Medical Association, N. S. Davis, M.D., now of the Rush Medical College, Chicago, has presented another great thought for consideration, which should command the attention of those who deplore the increase of quackery in these United States. Because the expense of a medical education is now burthensome, and annually becoming more so, interlopers are multiplying, and fast gaining in numbers and resources; in short, quackery of every possible grade thrives exc edingly. A mortifying fact in connection with this state of things is, that the people everywhere patronize this class of practitioners, notwithstanding their ignorance, misrepresentations and utter incapacity in the management of diseases. All this is matter of universal notoriety; but it is useless to apologize for those intelligent persons in the community who countenance such speculations on health, or to reason with any class of people upon the evil consequences that have an origin from this prolific source. Quackery exists, and stares us boldly and unblushingly in the face---and the question is, What is to be done? Mr. Davis says free medical schools must be organized. Here is his own language, cogent and truthful too. Speaking of the expensive character of the schools in general, he says---"It contributes to the general prevalence of quackery, by inducing many, who, in a free country, are determined to be doctors at all hazards, to embrace some of the numerous special systems that can be learned in a week or a month, instead of attempting to encounter the embarrassments of a full and regular course. And that it greatly limits the amount of medical knowledge and consequent skill, which a very large proportion of those who enter the profession are desirous of obtaining, is too evident to require a word. Hence, in whatever aspect we view the subject, candor compels us to acknowledge that the present system of medical instruction is alike injurious to the schools, unjust and embarrassing to the profession, and greatly detrimental to the best interests of the whole community." In accordance with the doctrine he inculcates, three of the faculty of the Rush Medical College give their lectures entirely free of charge to all the regularly matriculated students of that institution. It will soon become Rush College, in more senses than one---for there will be an avalanche of young men rushing to Chicago, where knowledge is had for asking; like the tide of emigrants to California, where gold is supposed to be had by simply picking it up.

We foresee that this extraordinary development will lead to a revolutionary movement in places which have been considered as firm as the hills. A modifying policy must be pursued, or there will be smaller classes in other schools, when this new idea is thoroughly abroad, than have been known since the days of their formation. Young gentlemen may go to Chicago, and have all and every advantage which a thrifty, chartered college can command, without money and without price. On this new plan, neither begging, nor certificates of poverty, are to be required to gain admission. The rich and the poor will be on the same level---they may hear, see and improve, without paying or feeling under obligations either to the faculty or the State.

